# The Dynamic Computer Workstation—A Pilot Study of Clinical and Biochemical Investigation during Work at Static Respectively Mobile Keyboards

**DOI:** 10.3390/ijerph18041493

**Published:** 2021-02-04

**Authors:** Bijar Ghafouri, Karin Wåhlén, Ulrika Wentzel-Olausson, Staffan Smeds

**Affiliations:** 1 Pain and Rehabilitation Center, and Department of Health, Medicine and Caring Sciences, Linköping University, 58183 Linköping, Sweden; karin.wahlen@liu.se (K.W.); ulrika.wentzel.olausson@liu.se (U.W.-O.); 2 Department of Health, Medicine and Caring Sciences, Linköping University, 58183 Linköping, Sweden; staffan.smeds@dynatab.se

**Keywords:** chronic pain, neck/shoulder pain, biomarkers, computer working, work-related neckpain

## Abstract

A large and increasing number of the work force in the population spend their work hours at the keyboard. There is evidence that repetitive high levels of static work, or extreme working postures involving the neck–shoulder muscles are an increased risk for chronic neck–shoulder pain. The aim of this study was to investigate the effect of dynamic computer working (DCW), using a mobile application to the desk surface, on pain characteristics and biomarkers in office workers. We included 10 female subjects. All subjects answered questionnaires about general health, pain intensity and characteristics. The pressure pain threshold (PPT), neck range and motion, neck and shoulder strength were measured. Microdialysis was conducted in trapezius muscle. Measurements were performed before and 4 weeks after DCW. Multivariate analysis, orthogonal partial least square discriminate analysis (OPLS-DA) and univariate analysis paired test, Wilcoxon, was performed. There was significant improvement in reported neck pain, quality of life, and psychological distress after 4 weeks DCW. The PPT and strength in neck and shoulder were significantly increased after DCW. A significant OPLS-DA model showed clear separation between the samples collected before and after 4 weeks DCW. In conclusion, these results show that keyboard work at a movable desk application might decrease the risk of repetitive strain injuries in the neck and shoulder muscles.

## 1. Introduction

Exposure to repetitive work and awkward working positions are known risk factors for neck and neck–shoulder musculoskeletal disorders (MSD) that is common in several professions including office workers using computers [1,2]. Repetitive work includes activities that involve continuous arm or hand movements which affect the neck–shoulder musculatures and generate load on the trapezius muscles [3,4,5].

The prevalence of reported neck and shoulder pain is 10–20% and affects women in a higher manner [6,7]. A large and increasing number of the work force in the population spend their work hours at the keyboard. This work situation implies, if static, a restricted position of the hands over the keyboard and narrow the hand movements in relation to the other parts of the upper extremity and stabilizing areas in neck and back muscles [8]. This, in turn, implies a non-physiological stress in the most easily excitable muscle fibers [9] in the common work situation. More severely, it has been reported that a significant fraction of the work force develops pain symptoms in the neck region, shoulders, and arms which may hamper effectivity and lead to sick leave [10].

This work-related problem has led to increasing interest in attempts to understand the biochemical background behind the pain signaling with focus on identification of specific molecular patterns during an induced pain situation [11,12]. There have been several studies investigating the structure and biochemistry of the myalgic trapezius muscle (TM) and increased type I muscle fibers were found [13,14]. Patients with a high pain score had not only the lowest capillary to fiber area ratio for type I fibers but also the proportion of cytochrome c oxidase negative fibers was higher in patients with more evolved TM [13]. This together with the low capillary to fiber areas indicates an energy crisis within the muscle and might affect the oxygen delivery to the muscle cells. Furthermore, it has been reported that impairment of metabolites in the working muscle is associated with painful trapezius muscles [13,15]. There have also been reports of mitochondrial disturbances known as moth eaten and ragged red fibers [16]. Microdialysis is a commonly used sampling method for in vivo studies which has been used to investigate the molecular alterations in the trapezius muscle. Increased concentrations of pyruvate, lactate, serotonin, and glutamate which is a strong indication of alterations in energy metabolism have been reported in several studies using microdialysis [17,18,19].

The invention of an office table at which the keyboard is situated on a slowly moving part of the desk (DynaDesk), gives the opportunity to study to what extent a mobile work situation will influence both the experience of as well as the biochemical variation in a dynamic work situation. The present study encounters self-reported assessment and physiological tests as well as microdialysis sampling of intramuscular biochemical markers, compared in individuals working in both the static and dynamic work situation.

## 2. Materials and Methods

### 2.1. Subjects

A total of 10 women who worked 60% (minimum 4.8 h per day for a full-time worker) of their work time at a computer were recruited via local advertisement in neighboring workplaces. The demographic data is presented in Table 1.

### 2.2. Ethics

The study was granted ethical clearance by Linköping University Ethics Committee (Dnr: 2019-03482). All participants gave their written informed consent, and the study was performed in accordance with the Helsinki Declaration.

### 2.3. Procedure

At the first visit, the participants underwent a clinical examination including pain sensitivity testing and physical tests. In immediate connection to the first visit, the subjects answered a health questionnaire covering demographic data as well as pain aspects, psychological characteristics, and health aspects. These questionnaires have been used in studies investigating chronic pain in our previously published works. The subjects were instructed to not perform strenuous exercise two days before, and not to take any pain killer medication to decrease any effects on the biomarkers and potential complication due to the microdialysis catheter insertion on the muscle. Microdialysis of the trapezius muscles was performed at the second visit a few days later. The participants were instructed how to use DynaDesk (WorkMotions, Stockholm, Sweden) and each test person was assigned a DynaDesk at their ordinary working place without change of light or chair. Microdialysis of the trapezius muscles during static computer work (SCW) was performed at the second visit a few days later. After 4 weeks of dynamic computer working (DCW) in their ordinary office work situation, the subjects answered the questionnaires and all tests, pain sensitivity, physical test and microdialysis were repeated. The subjects were allowed to perform their daily activity or exercise as usual and were asked to not change any activities during these 4 weeks.

### 2.4. The Dynamic Desk DynaDesk

**Figure 1:**
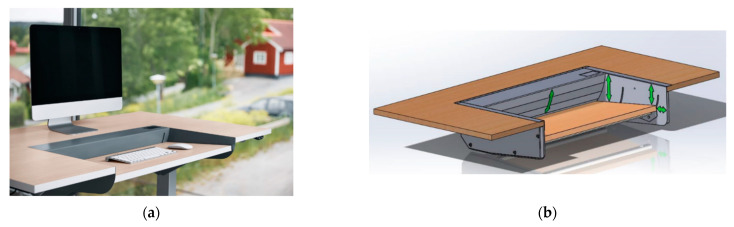
(**a**). An illustration of the dynamic desk DynaDesk. (**b**). Schematic pattern which demonstrates the three movements as executed by DynaDesk: Forward-backward, down-up and forward tilting.

DynaDesk has a rectangular moving tray on which the keyboard and mouse are placed as shown in Figure 1a. The tray moves continuously in a slow dynamic pattern–downwards, forwards, slightly tilting, and then back again to the starting point, see Figure 1b. The continuous movement is soft, and the flow rate can be adjusted individually. In the standard version which has been used in this study, the moving DynaDesk keyboard tray is centered along the side of the desk and designed as a rectangular cut-out in the work surface. The keyboard tray is approximately 75 cm wide and 35 cm deep. On each side of the DynaDesk tray is the ordinary fixed work surface of the desk. In addition to the perpetual movement of the keyboard tray along a forward and downward slope, it was observed by surface electromyographical analyses that forward tilting of the tray was necessary to neutralize an observed increased muscular activity in the extensor carpi muscles when the tray remained horizontal during the work process. DynaDesk is patented [20].

### 2.5. Questionnaires

All subjects answered a brief questionnaire including information about age (years), aspects of pain (intensity and duration), psychological distress, and health aspects.

The pain intensity in neck, shoulder, arm, head, feet, and hand during recent 7 days was reported using a Numeric Rating Scale (NRS) from 0 (= no pain) to 10 (= worst possible pain) [21,22].

The Hospital Anxiety and Depression Scale (HADS) was used for measuring the psychological distress. The subscales HADS-depression and HADS-anxiety have seven items, scoring range between 0 and 21, in which a lower score indicates a lower possibility of anxiety or depression. HADS is frequently used in clinical practice and research and has good psychometric characteristics [23,24].

The Quality-of-Life Scale (QOLS) is a 16-item instrument that measures quality of life which includes material and physical well-being, relationships with other people, social, community and civic activities, personal development, and fulfillment. The QOLS is scored by adding up the score on each item to yield a total score for the instrument. Scores can range from 16 to 112 [25].

### 2.6. Pressure Pain Thresholds

A handheld manual electronic pressure algometer (Somedic, Hörby, Sweden) was used to assess pressure pain thresholds (PPT) [26]. Pressure was measured in kilopascal (kPa). The participants were instructed about the procedure before the actual testing. The pressure was applied at a rate of 30 kPa/s, with a 1-cm diameter probe. All participants were instructed to press a button when they felt the first sensation of pain, not merely pressure. The maximum pressure was set at 600 kPa, at which point the application of pressure ceased. The test sites were located at three points along the upper part of the trapezius muscle, bilaterally, and at three points over the belly of the Tibialis anterior muscle, bilaterally. The Tibialis anterior muscle was used as control muscle.

### 2.7. Physical Function Tests

#### 2.7.1. Strength

Maximal isometric neck strength was measured with a handheld dynamometer [27,28,29]. The force was measured in Newton. Neck extension was assessed with the body lying in a prone position on an examining table with legs straight and arms alongside the body [30]. The head was hold in a maximal extension without contact with the examining table. The instruction to the subject was to hold the head still (–“hold still-don’t let me push your head!”) when the examiner (tester) gradually put an increasing pressure on the back of the head until the head position (the force) was impossible to maintain. The procedure was repeated three times.

Neck flexion was assessed with the body lying on the examining table in a supine position with straight legs and arms alongside the body [31]. The head was hold in a flexed position without contact with the examining table. The chin was kept as close as possible to the chest. The instruction to the subject was to hold the head still (–“Hold still, don´t let me push your head!”) when the tester applied gradually increasing pressure on the forehead until the position or force could no longer be maintained. The procedure was repeated three times.

Dynamic strength in the shoulders was assessed [31] in a standing position by counting the number of repetitions holding a pair of 3 kg dumbbells. There were two different movements performed, shoulder abduction with straight arms and upright row. The instruction was to perform as many repetitions as possible. The test was interrupted when the participants failed to do the exercise correctly due to fatigue or pain.

#### 2.7.2. Neck Range of Motion

Active mobility in the neck was evaluated in a sitting position with arms alongside the body, and the trunk and head hold in a straight or upright position. Active range of motion was measured in the sagittal, frontal, and transvers planes. The cervical mobility instrument that was used in this study is a validated and reliable tool [32], consists of a helmet with two gravity goniometers [33], two spirit levels, and a compass to evaluate flexion, extension, lateral flexion, and rotation of the neck. The participants were instructed to keep the thorax in the same position and only move the cervical part of the spine [31]. There was no encouragement from the tester during the procedure.

### 2.8. Microdialysis

Microdialysis (MD) sampling devise allows investigation of local biochemical changes in the trapezius muscle interstitial compartment before dilution in the vascular compartment and systemic. It consists of a thin semi permeable catheter, mimicking a blood vessel, which is inserted in the tissue. The catheter is infused with physiological saline (the perfusate) by a mechanical pump and by diffusion, down a concentration gradient, and collects the substances that pass through the catheter membrane [34,35,36].

The subjects were instructed not to perform strenuous exercise two days before the study, not to drink any beverages with caffeine and not to smoke on the day of the study. In addition, they were asked not to take paracetamol-medication two days before and NSAID (Non-Steroid Anti-Inflammatory Drug) -medication one week before the MD sessions.

Before the insertion of the catheter, ultrasound measurement was performed on the dominant trapezius muscle to determine the distance between the skin and the muscle and the width of the muscle to guide the insertion of the catheter. Using the SENIAM (surface electromyography for a non-invasive assessment of muscles) landmarks [37], the midpoint of the line between the spine of 7th cervical vertebra and the acromion was defined as the midpoint of the descending trapezius. The skin and subcutaneous tissue at the catheter entrance was anesthetized with a local injection (0.5 mL) of lidocaine (Xylocain^®^ 20 mg/mL, AstraZeneca, Södertälje, Sweden) without adrenaline. Care was taken not to anesthetize the underlying muscle. Commercially available MD catheter (CMA 71, CMA Microdialysis AB, Solna, Sweden)–cut-off point of 100 kDa, membrane 30-mm length, 0.5-mm diameter–was inserted parallel to the muscle fibers into the trapezius muscle. The catheter was inserted in the middle third of the upper part of the trapezius muscle in lateral to medial direction. The catheter was perfused with a high-precision syringe pump (CMA 107; CMA/Microdialysis AB, Solna, Sweden) at a rate of 5 μL/min with the Perfusion Fluid T1 (P000034, Microdialysis AB, Solna, Sweden).

**Figure 2:**
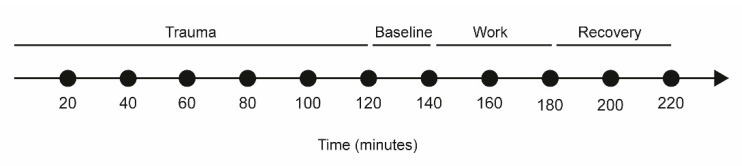
Timeline of microdialysis sampling. The time 0–120 min refer to tissue trauma, 120–140 min refer to baseline, 140–180 min is the working period and 180–220 is called the recovery period.

Immediately after the insertion of catheters, participants rested comfortably in an armchair for 120 min (i.e., the trauma period) to allow the tissue to recover from possible changes in the interstitial environment induced. The response to implantation of a microdialysis catheter can broadly be classified as; trauma caused by guide needle or catheter insertion [38]. Groth et al. [39] have suggested that an equilibration period of 90–120 min is required after catheter insertion in human skin. The trauma period of 120 min has been used in microdialysis experiment in trapezius muscle in human [40]. After the trauma period, participants continued to rest for 20 min, the baseline period (denoted 140 min) [19,40]. The baseline period was followed by a 40-min period of computer working. The work assignment consisted of writing a standardized text during the 40 min. The experiment ended with a recovery period of 40 min during which participants rested in the armchair, a schematic illustration of the time periods is shown in Figure 2.

During the MD session, subjects rated the pain intensity in trapezius muscle in right and left side, every 20 min using NRS. Samples were obtained every 20 min during the in total 220 min long MD test period and the samples were kept on ice throughout the MD experiment. The samples were then stored as aliquots in −86 °C until analysis. Vials with visible sign of hemolysis were discarded.

### 2.9. Biochemical Analysis

The dialysate samples from baseline, work period, and recovery were thawed, centrifuged, and analyzed for the concentrations of pyruvate, lactate, glutamate, glycerol, and glucose with ISCUSflex Analyzer (CMA Microdialysis AB, standard range, Solna, Sweden). The detection intervals are as follows: 0.1–12 mmol/L for lactate; 10–1500 µmol/L for pyruvate; 1.0–150 µmol/L for glutamate; 0.1–25 mmol/L for glucose; and 10–1500 mmol/L for glycerol.

For measuring the concentration of inflammatory proteins, a commercially available 71-panel of cytokines/chemokines (No. K15081K-1) from MesoScale Discovery (Meso Scale Diagnostics, Rockville, MD, USA) was used according to the manufacturer’s instructions.

### 2.10. Statistics

For analysis of demographic data, the median value (min-max) was calculated. Wilcoxon paired test was used to compare results before and after the intervention. These statistics were performed using the statistical package IBM SPSS Statistics (version 24.0; IBM Corporation, Route 100 Somers, New York, NY, USA). A probability of <0.05 (two-tailed) was accepted as the criteria for significance. To investigate the multivariate correlation and between membership of groups and quantified proteins and metabolites orthogonal partial least square discriminant analysis (OPLS-DA) was used (SIMCA-P+ version 13.0, UMETRICS, Umeå, Sweden). Before this analysis, principal component analysis (PCA) was used to check for multivariate outliers. The procedure to compute multivariate correlation models has been described earlier [41] and is in accordance with [42].

## 3. Results

The demographic data is presented in Table 1. Data from one subject was excluded from the analysis due to defect of the DynaDesk. The moveable part of the desk was unstable, and the subject decided to not use the movable function of the desk. The dialysate samples from each 20-min sampling were analyzed for the metabolites (pyruvate, lactate, glucose, glycerol, and glutamate). In total 198 samples at static computer work (SCW) and DCW were analyzed. For protein analysis pooled samples from different timepoints were used due to low recovery of proteins. The samples from each subject were pooled individually as: timepoint 0–100 (trauma), 120–140 (baseline), 160–180 (working), and 200–220 (recovery) (Figure 2). In total 72 samples were analyzed.

### 3.1. Self-Reported Markers

**Figure 3:**
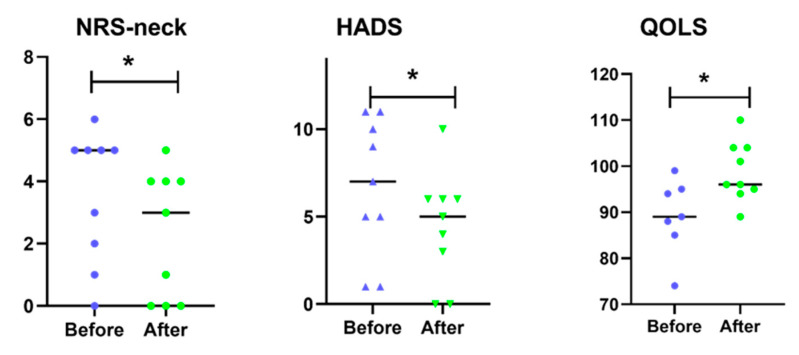
Statistical analysis of self-reported markers. Numeric rate scales in the neck (NRS-neck), hospital anxiety and depression scale (HADS), and quality of life scale (QOLS) for all subjects are presented in the figure. * indicates statistical significance (*p* < 0.05) differences between before dynamic computer working (DCW) (blue) and after 4-weeks DCW (green) using DynaDesk. The median of the data is marked in the figure.

There was significant reduction (*p* < 0.05) in pain in the neck after 4-weeks DCW (Figure 3). As illustrated in Figure 3, subjects reported significantly lower HADS after 4-weeks DCW. The quality-of-life scale was significantly higher after 4-weeks DCW compared to before DCW (Figure 3).

### 3.2. Physiological Markers

**Figure 4:**
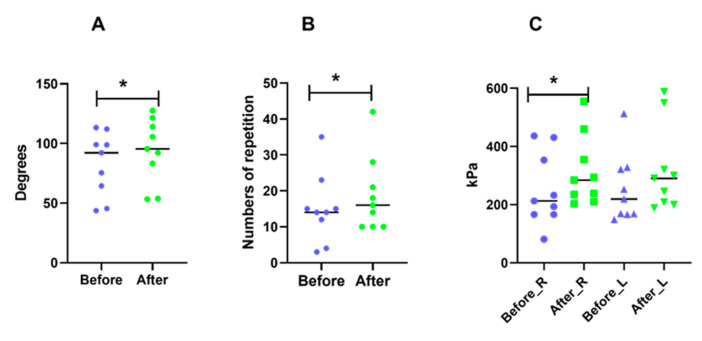
Statistical analysis of physiological markers Neck strength (**A**), shoulder strength (**B**) and pain pressure threshold (PPT) (**C**) for all subjects are shown. * indicates statistical significance (*p* < 0.05) differences between before dynamic computer working (DCW) (blue dots) and after 4-weeks DCW (green dots) using DynaDesk. R refers to right side trapezius muscle and L refers to left side trapezius muscle. The median of the data is marked in the figure.

There was significant improvement (*p* < 0.05) for neck and shoulder strength (Figure 4A,B) after DCW. The pain sensitivity was significantly decreased in the dominant trapezius, the same trend was observed for the left side but did not reached statistical significance (Figure 4C).

### 3.3. Biological Markers

**Figure 5:**
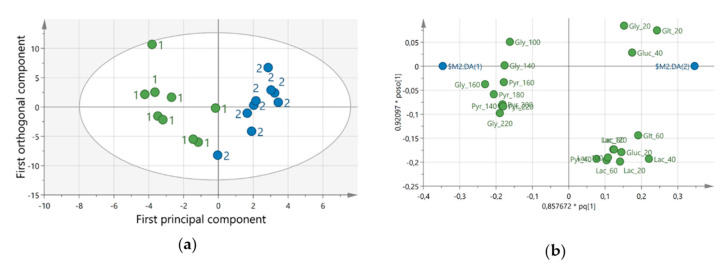
Multivariate data analysis of the metabolite. (**a**) represents the score plot of the orthogonal partial least square discriminant analysis (OPLS-DA) showing a clear separation between samples from before (green circles) dynamic computer working (DCW) and after (blue circles) 4-weeks DCW using DynaDesk. (**b**) illustrates the loading plot for the OPLS-DA model showing the substances (Gly = glycerol, Glt = glutamate, Pyr = pyruvate, Lac = lactate, Gluc = glucose) that are important for the separation between the groups. The substances are listed in Supplementary Table S1.

**Figure 6:**
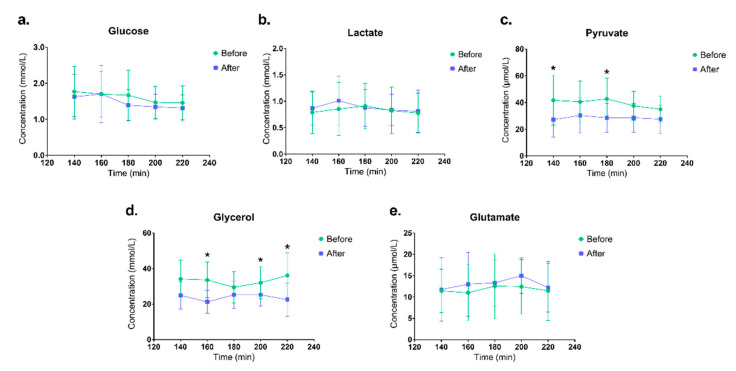
An overview of the metabolite concentrations over time (**a**) Glucose concentrations; (**b**) Lactate concentrations; (**c**) Pyruvate concentrations; (**d**) Glycerol concentrations; (**e**) Glutamate concentrations. The concentrations of the metabolites at baseline (140 min), working period (160–180 min) and recovery period (200–220 min) are shown as mean value and the bars represent standard deviation. * indicates a statistical significant (*p* < 0.05) differences between samples before dynamic computer working (DCW) and after 4 -weeks DCW.

Data for the metabolites were analyzed by multivariate data analysis (MVDA). A significant OPLS-DA model (CV-ANOVA = 0.044, R2 = 0.81, Q2 = 0.52) could separate the samples before and after 4-weeks of DCW. The score plot and loading plot are shown in Figure 5. In Supplementary Table S1 the important variables that contribute to the separation between the samples are presented. The concentrations of metabolites at trauma period (0–100 min) seems to be more associated with samples collected at SCW and data from the working period and recovery (160–220 min) were more associated to samples at DCW. An overview of the metabolite concentrations over time is presented in Figure 6. A decreased level of pyruvate and glycerol was detected after 4-weeks DCW. The decreased concentrations for pyruvate at baseline and work period and glycerol at work and recovery period were statistically significant (*p* < 0.05).

**Figure 7:**
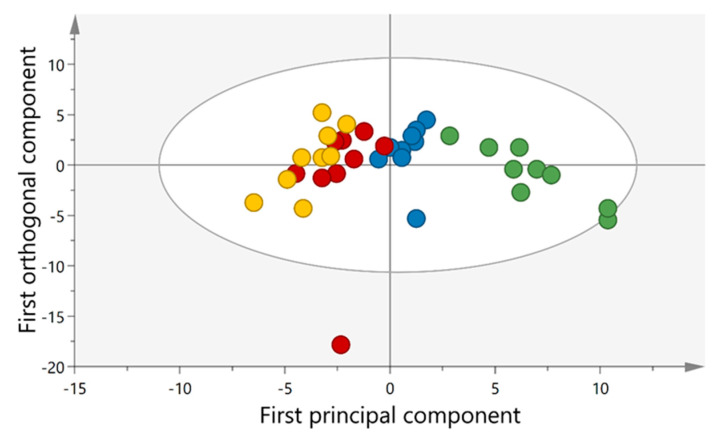
Score plot of the orthogonal partial least square discriminant analysis (OPLS-DA) model for cytokines and chemokines showing separation between the samples from trauma (green circles), baseline (blue circles), work (red circles) and recovery (yellow circles) periods.

The levels of 56 of the cytokines/chemokines were detectable and included in the statistical analysis. A significant OPLS-DA (CV-ANOVA = 0.011, R2 = 0.31, Q2 = 0.24) model could separate the samples from different time points; trauma, baseline, work, and recovery (Figure 7). The separation was based on 31 contributing proteins with VIP (variable of importance) > 1 and *p*(corr) > 0.3 that are listed in Supplementary Table S2.

**Figure 8:**
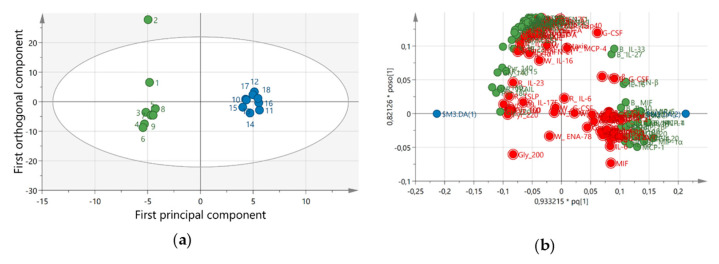
Score plot of the orthogonal partial least square discriminant analysis (OPLS-DA) model (**a**) and loading plot (**b**) for all data. The score plot shows a clear separation between samples dynamic computer working (DCW) (green circle) and after 4-weeks DCW (blue circle). The loading plot represents the substances that contribute to the separation between the groups. The important variables with variable of importance (VIP) > 1 are marked green. Red marked variables have a VIP < 1 and is regarded as non-significant analytes.

All biological data including the self-reported and physiological markers were subjected to MVDA and a significant OPLS-DA model (CV ANOVA = 0.003, R2 = 0.87, Q2 = 0.69) was achieved (Figure 8). The important variables (VIP > 1 and *p*(corr) > 0.3) that contribute to the separation is listed in Supplementary Table S3.

## 4. Discussion

This study aims to investigate if dynamic computer working (DCW), as conducted with the DynaDesk application, can reduce work-related musculoskeletal disorders (MSD) of the neck and shoulder compared to a static computer work (SCW). The major findings in this intervention study are:

1. Significant decreased self-assessments of neck pain, psychological distress, and increased quality of life were found after 4-weeks of DCW.
2. Significant increased pressure pain threshold and neck and shoulder strength were found after 4-weeks of DCW.
3. Decreased levels of pyruvate, glycerol, and inflammatory proteins were found during DCW.

While the etiologic mechanisms behind chronic neck and shoulder pain are poorly understood [12,17,40], there is increasing evidence that psychosocial factors related to the job and work environment play a role in the development of MSD [43]. In this study we found significantly decreased psychological distress and improved quality of life in parallel with less self-assessed pain in the neck when the test persons performed computer work using the dynamic desk. The pain sensitivity in the trapezius muscle and the strength in neck and shoulder were also significantly improved. These findings support the importance of psychosocial factors in chronic pain. There is evidence that repetitive high levels of static work, prolonged static loads, or extreme working postures involving the neck–shoulder muscles increase the risk for development of chronic neck–shoulder pain [44]. The trapezius muscle is often involved in neck–shoulder pain, but it’s pain pathophysiology is not fully elucidated, and several hypotheses have been suggested [12]. Microdialysis studies have previously been used to investigate biochemical alterations in trapezius muscle in patients with chronic muscle pain [45,46]. In patients with chronic trapezius myalgia, this approach has shown that the metabolic responses to brief exercise are abnormal [18]. When compared to healthy controls, patients with chronic neck pain [47] and myofascial trigger points [48] have been shown to have higher interstitial concentrations of several substances. Thus, increased level of pyruvate has previously been reported in patients with trapezius myalgia and patients with fibromyalgia compared to healthy controls [35,49,50]. The subjects included in the present study have not been diagnosed as patient with chronic pain. In accordance with previous findings, the present subjects without chronic pain have been identified with higher concentration of metabolites during SCW compared to DCW. This may indicate that SCW can give rise to disturbed muscle energy metabolism without development or signs of chronic pain. The reason behind the development of chronic pain or not despite disturbed electrolyte metabolism in individual subjects is not known but the present findings indicate a risk situation during static computer work. Decreased levels of pyruvate and glycerol was observed after 4-weeks DCW. Pyruvate is the key product of the glycolysis that produce glucose and glycogen and it can be reduced to lactate. There is evidence that under aerobic conditions lactate is produced and transported to the mitochondria to be converted to pyruvate resulting in ATP (adenosine triphosphate) production [51,52]. The observed decreased levels of pyruvate after DCW might indicate an increased energy production in the muscle and, hypothetically, less cell injury. Glycerol has been reported as a marker for cell injuries [53] as produced by hydrolysis of triglycerides of muscle cells [54]. Increased levels of muscle glycerol has been shown in patients with trapezius myalgia [46]. We have found decreased levels of glycerol after DCW compared to the static work situation. It has previously been reported that the increased levels of pyruvate might be a result of an ongoing inflammation that prevents the metabolism of pyruvate to produce energy [55]. Interestingly, we found decreased levels of several inflammatory proteins after DCW. This indicates that a decreased inflammatory environment in the muscle possibly results in less injured muscle tissue and augmented production of sufficient energy for the contracting muscle fibers. The automated movement with the hands following the movable keyboard may also, hypothetically, change the region in the muscle where the most excitable muscle fibers are activated. Alternating the contraction pattern in muscles of the upper limb, albeit very small, may hypothetically also influence the microcirculation. 

There are several limitations in this study. The present study was performed on female subjects recruited in the neighboring workplaces and the findings can therefore not be generalized to all keyboard workers. Neither can the results indicate any age group with augmented propensity for developing computer work induced pain in the neck–shoulder muscles. Another limitation was the relatively low number of included subjects. Microdialysis sampling is an invasive and time-consuming technique which limit the recruiting of subjects. Future studies including larger cohort containing a group diagnosed with chronic neck–shoulder pain and a group without any diagnosis of chronic pain are highly recommended. The lack of control for daily activities and exercise during the 4 weeks of the investigation is another limitation of this study. It remains to be analyzed if the observed physical and biochemical findings are general findings during dynamic computer work and if an alleviation as observed can be expected in a lager range of computer workers. Future studies are warranted to elucidate these questions.

## 5. Conclusions

The biochemical analyses indicated individual substances of importance for the difference between static and dynamic work and their respective variation in concentration during the MD period. Furthermore, temporal analysis of the significant substances as observed, may give an increased understanding of possible interaction pattern during low-intensive static muscle load. It remains also to study whether DCW will alleviate pain in patients with MSD. Taken together, the present pilot findings give evidence that the ergonomic desk decrease the risk of repetitive strain injuries in the neck–shoulder muscles.

## Figures and Tables

**Table 1:** Background data.

	Subjects (%) *n* = 9	Median (Min-Max)
Age		54 (29–73)
Length (cm)		167 (153–178)
Weight (Kg)		69 (50–84)
BMI		24 (21–31)
Education		
High school	2 (22)	
University	7 (78)	
Profession		
PhD student	1 (11)	
Research nurse	1 (11)	
Lecturer	2 (22)	
Administrator	2 (22)	
Statistician	3 (33)	
Pain		
Duration (years)	32 (11), 15 (11), 14 (22), 10 (11), 9 (11), 4 (11)	
Continuously	2 (22)	
Periodical	5 (55)	
Medication	3 (33)	
Smoking		
Non-smoker	6 (66)	
Former smoker	3 (33)	

BMI: Body Mass Index.

## Data Availability

The data are available on request from the corresponding author.

## Supplementary Materials

The following are available online at https://www.mdpi.com/1660-4601/18/4/1493/s1, Table S1: Multivariate statistics for metabolite concentrations in microdialysis samples before and after 4-weeks dynamic computer working (DCW) using DynaDesk. Table S2: Multivariate statistics for cytokines/chemokines concentrations in microdialysis samples during static computer working (SCW) and after 4-weeks dynamics computer working (DCW) using DynaDesk, Table S3: Important variables for separation between samples collected at static computer working (SCW) and 4-weeks after dynamic computer working (DCW).
