# Supplementary material for: The Dynamic Computer Workstation—A Pilot Study of Clinical and Biochemical Investigation during Work at Static Respectively Mobile Keyboards
Source: Int J Environ Res Public Health. 2021 Feb 4;18(4):1493. doi: 10.3390/ijerph18041493 (PMC7915059; doi:10.3390/ijerph18041493)
Supplement: Supplementary file 1 [file ijerph-18-01493-s001.pdf]

Table 1. Multivariate statistics for metabolite concentrations in microdialysis samples before and after 4-weeks dynamic computer working (DCW) using DynaDesk. The name of the metabolites and corresponding time points are listed under Substances. VIP (variable of importance), the sign of p(corr) refers to up (+) and down (-) regulated substances comparing samples from before and after DCW. The concentrations are in mmol/l for LAC (lactate), GLT (glutamate), GLY (glycerol), GLUC (glucose) and µmol/l for PYR (pyruvate) and the mean ± standard deviation (SD) is listed.

SUBSTANCES	VIP	P(CORR)	[BEFORE] MEAN (SD)	[AFTER] MEAN (SD)
**LAC_40**	1.72	0.45	0.58 (0.25)	0.86 (0.36)
**GLT_20**	1.68	0.76	40.57 (9.81)	68.00 (21.37)
**GLY_160**	1.57	-0.73	33.55 (10.03)	21.22 (6.41)
**GLT_60**	1.45	0.61	14.11 (7.80)	23.33 (7.36)
**PYR_180**	1.43	-0.66	42.66 (15.48)	28.55 (10.85)
**GLY_220**	1.36	-0.6	36.11 (12.55)	22.44 (9.23)
**PYR_140**	1.31	-0.59	41.66 (18.65)	27.22 (13.27)
**PYR_200**	1.29	-0.58	37.55 (10.84)	28.55 (10.72)
**PYR_220**	1.28	-0.57	34.88 (9.70)	27.55 (10.59)
**PYR_160**	1.22	-0.57	40.55 (15.44)	30.33 (13.27)
**GLY_140**	1.2	-0.57	34.11 (10.63)	24.88 (7.60)
**GLUC_40**	1.18	0.53	1.10 (0.31)	1.54 (0.29)
**LAC_120**	1.14	0.39	0.66 (0.33)	0.91 (0.35)
**LAC_80**	1.13	0.39	0.62 (0.36)	0.87 (0.44)
**GLY_100**	1.13	-0.52	47.00 (17.66)	34.77 (10.68)
**LAC_60**	1.12	0.33	0.62 (0.32)	0.93 (0.56)
**LAC_100**	1.11	0.34	0.64 (0.29)	0.92 (0.42)
**GLY_20**	1.1	0.52	51.14 (16.06)	80.22 (19.67)

Table 2. Multivariate statistics for cytokines/chemokines concentrations in microdialysis samples during static computer working (SCW) and after 4-weeks dynamics computer working (DCW) using DynaDesk. The substances are ordered from highest VIP (variable of importance), the sign of p(corr) refer to up (+) and down (-) regulated substances comparing the groups. The concentrations are in pg/ml. Missing data mean analyte was not analysed at the specific time point. Substances are presented as mean ± standard deviation (SD).

			SCW				***DCW***			
Substances	**VIP**	**p(corr)**	**Trauma**	**Baseline**	**Work**	**Recovery**	**Trauma**	**Baseline**	**Work**	**Recovery**
IL-17D	1.65	0.81	48.95 (43.88)	85.23 (135.32)	33.70 (47.66)	18.36 (22.66)	46.00 (13.10)	33.65 (17.75)	14.92 (12.41)	5.95 (9.02)
IL-18	1.63	0.80	2.05 (1.11)	1.49 (1.15)	2.12. (2.80)	1.46. (0.86)	3.49 (1.47)	1.08 (0.53)	0.88 (0.36)	0.87 (0.24)
IL-1β	1.58	-0.66	0.03 (0.05)	0.09 (0.11)	0.21 (0.31)	0.22 (0.19)	0.03 (0.01)	0.12 (0.07)	0.21 (0.08)	0.24 (0.14)
MIP-5	1.54	0.75	95.62 (90.28)	19.24 (14.31)	37.88 (30.56)	28.69 (21.18)	222.01 (136.93)	41.24 (30.10)	36.47 (22.46)	32.55 (22.42)
IL-29/IFN-L1	1.49	-0.66	0.01 (0.02)	4.70 (5.89)	5.15 (5.23)	3.58 (2.09)	0.55 (0.33)	2.50 (1.14)	3.31 (0.63)	2.55 (0.70)
IL-8	1.48	-0.63	4.39 (6.50)	17.14 (23.21)	74.57 (107.47)	63.20 (70.11)	7.73 (4.83)	32.18 (20.49)	59.05 (36.19)	60.22 (39.04)
VEGF-A	1.44	0.70	2.52 (2.81)	0.00 (0.00)	0.60 (1.38)	1.09 (2.18)	2.53 (1.77)	0.12 (0.35)	0.21 (0.46)	0.25 (0.74)
MCP-1	1.43	-0.67	59.10 (50.80)	288.48 (179.99)	769.36 (588.38	827.52 (521.03)	106.99 (38.07)	562.68 (323.55)	910.98 (420.99)	1093.78 (722.04)
MDC	1.38	-0.42	3.91 (5.34)	4.14 (7.33)	34.54 (36.17)	19.97 (18.75)	5.32 (6.57)	8.56 (10.19)	17.20 (15.56)	16.18 (16.72)
MCP-4	1.38	-0.50	2.38 (2.61)	1.39 (2.03)	10.91 (7.29)	10.58 (9.53)	6.10 (2.80)	5.87 (4.04)	10.08 (6.77)	12.84 (14.61)
IL-10	1.30	-0.47	0.001 (0.001)	0.001 (0.004)	0.01 (0.01)	0.02 (0.02)	0.01 (0.01)	0.01 (0.01)	0.01 (0.01)	0.01 (0.01)
IL-17A/F	1.30	-0.44	0.22 (0.32)	0.64 (0.67)	2.41 (2.32)	1.69 (1.26)	0.62 (0.58)	1.51 (1.05)	1.56 (1.29)	1.56 (0.99)
IL-1RA	1.27	-0.57	75.98 (3.44)	156.85 (111.80)	278.77 (254.48)	357.23 (216.83)	188.32 (122.53)	298.96 (157.08)	349.42 (133.43)	513.49 (290.02)
IFN-α2a	1.26	-0.62	0.49 (0.33)	1.02 (1.16)	1.53 (1.68)	1.06 (0.52)	0.34 (0.25)	0.50 (0.33)	0.62 (0.39)	1.01 (0.20)
IL-16	1.25	-0.33	2.71 (2.20)	3.33 (4.19)	6.30 (5.12)	5.81 (4.63)	5.01 (3.44)	5.06 (2.71)	4.64 (1.70)	5.99 (4.01)
IL-22	1.23	-0.60	1.17 (1.43)	2.11 (2.84)	1.98 (2.06)	1.23 (0.53)	0.60 (0.09)	1.01 (0.30)	1.04 (0.16)	0.98 (0.23)

IL-4	1.23	-0.54	0.00 (0.00)	0 .00 (0.00)	0.01 (0.01)	0.01 (0.01)		0.001 (0.002)	0.001 (0.01)	0.02 (0.01)
IFN-β	1.20	-0.60	4.97 (4.72)	7.06 (7.38)	39.67 (71.12)	33.96 (16.35)	8.95 (7.62)	16.85 (8.80)	24.52 (11.28)	42.92 (15.43)
MIF	1.17	0.56	30419.07 (23175.81)	6718.35 (528.15)	10716.26 (9984.92)	4714.79 (3516.22)	44575.21 (14007.22)	10783.91 (5240.24)	7796.39 (2652.24)	4347.19 (2707.24)
MIP-1β	1.16	-0.49	1.69 (1.75)	1.84 (1.86)	5.45 (3.89)	4.66 (3.34)	4.39 (3.59)	5.51 (4.27)	5.45 (3.17)	5.39 (4.47)
IL-6	1.15	-0.53	2.08 (2.27)	11.10 (11.55)	26.86 (26.80)	27.70 (23.17)	3.00 (1.50)	15.4586 (9.36)	21.61 (10.71)	21.96 (13.27)
ENA-78	1.14	0.47	0.91 (1.59)	0.01 (0.01)	0.08 (0.15)	0.08 (0.16)	1.67 (1.05)	0.01 (0.01)	0.04 (0.06)	0.08 (0.07)
IP-10	1.07	-0.45	0.99 (1.46)	0.27 (0.47)	3.91 (3.23)	7.70 (9.04)	3.21 (1.93)	2.08 (2.58)	7.18 (9.05)	19.31 (35.60)
M-CSF	1.06	0.35	81.46 (123.41)	5.54 (11.22)	43.10 (81.58)	10.92 (17.61)	31.55 (25.35)	6.67 (10.63)	4.45 (12.59)	11.87 (26.84)
TARC	1.06	0.49	1.37 (2.44)	0.30 (0.57)	1.02 (1.06)	0.72 (0.55)		0.68 (0.56)	1.03 (0.85)	0.92 (0.86)
IL-21	1.05	-0.44	1.03 (1.79)	3.67 (4.59)	12.99 (12.02)	11.56 (7.71)		9.00 (6.40)	11.46 (4.32)	8.39 (4.85)
MIP-1α	1.05	-0.45	6.07 (4.50)	3.56 (2.71)	12.06 (15.28)	8.77 (4.39)		6.99 (1.94)	8.93 (2.67)	10.88 (7.30)
G-CSF	1.04	-0.46	1.46 (1.60)	4.26 (4.90)	17.75 (16.56)	20.47 (28.60)		7.47 (6.88)	13.41 (12.03)	21.17 (26.18)
I-TAC	1.04	0.42	1.04 (1.66)	0.00 (0.00)	0.03 (0.09)	0.25 (0.59)	4.09 (8.13)	0.28 (0.81)	1.04 (2.26)	0.64 (0.95)
IL-1α	1.03	0.32	4.21 (5.02)	3.62 (4.49)	2.67 (2.94)	1.60 (0.66)	1.59 (1.18)	2.52 (2.32)	1.23 (0.29)	1.03 (0.42)
IL-33	1.03	0.30	7.45 (6.10)	1.44 (1.38)	4.93 (6.68)	2.74 (1.26)	15.67 (6.82)	2.41 (0.75)	2.55 (0.84)	2.49 (0.98)

Table 3. Important variables for separation between samples collected at static computer working (SCW) and 4-weeks after dynamic computer working (DCW). VIP= variable of importance. The sign of p(corr) refers to up (+) and down (-) regulated substances comparing SCW to DCW.B refers to samples collected at baseline, W at work period and R at recovery period.

VARIABLES	VIP	P(CORR)
GLT_20	1.56	0.63
B_ MCP-4	1.46	0.74
B_ MIP-1A	1.41	0.70
MCP-4	1.37	0.71
GLY_20	1.34	0.57
B_ MIP-5	1.32	0.69
B_ MCP-1	1.31	0.67
MCP-1	1.31	0.64
IP-10	1.29	0.67
IL-29/IFN-L1	1.28	0.75
MIP-5	1.26	0.64
B_ IL-1RA	1.26	0.66
IL-1RA	1.22	0.63
B_ IP-10	1.21	0.63
GLY_160	1.19	-0.56
IL-33	1.19	0.62
R_ IL-1A	1.18	-0.48
R_ MIP-3B	1.16	0.58
GLY_140	1.14	-0.53
W_ IL-3	1.14	-0.39
GLUC_40	1.13	0.49
B_ IFN-B	1.12	0.55
B_ IFN-Γ	1.12	0.57
R_ IL-18	1.12	-0.37
PYR_180	1.12	-0.55
W_ IFN-A2A	1.11	-0.37
R_ GM-CSF	1.10	-0.42
GLT_60	1.10	0.54
B_ MIP-1B	1.10	0.56
W_ IL-1A	1.09	-0.31
B_ IL-33	1.09	0.47
B_ IL-17C	1.09	-0.31
PYR_140	1.08	-0.49
IL-16	1.08	0.56
W_ TNF-B	1.08	-0.39
IL-18	1.08	0.56
B_ MIF	1.08	0.57
W_ IL-7	1.07	-0.32
TRAIL	1.07	-0.33
R_ TRAIL	1.04	-0.53
GLY_220	1.03	-0.50
B_ IL-27	1.02	0.45
B_ IFN-A2A	1.02	-0.31
R_ IL-22	1.02	-0.41
W_ M-CSF	1.02	-0.37
R_ IL-15	1.01	-0.48
R_ IL-1RA	1.01	0.55
IL-10	1.01	0.69
B_ IL-10	1.00	0.50
